# Validation and Psychometric Properties of the Italian Version of the Unconditional Self-Kindness Scale (USKS)

**DOI:** 10.3390/ijerph20105839

**Published:** 2023-05-16

**Authors:** Andrea Poli, Mario Miccoli

**Affiliations:** Department of Clinical and Experimental Medicine, University of Pisa, 56126 Pisa, Italy; andrea.poli@med.unipi.it

**Keywords:** self-kindness, mindfulness, compassion, psychological trauma, psychometrics

## Abstract

Western psychology and social sciences have long emphasized the value of a positive attitude toward oneself. Previous research had developed psychometric tools assessing self-compassion, defined as being open to and moved by one’s own suffering. However, self-compassion did not describe whether people actually applied such protective factors when acutely faced with threats. The Unconditional Self-Kindness Scale (USKS) was developed as a tool to measure the behavioral response of self-kindness during an acute presence of threat to the self and not just as a general attitude when threat is absent. Since it can be experienced even in the most challenging situations and may promote resilience, this kindness may be defined as unconditional. We validated the Italian version of the USKS and found that the scale retained a one-factor structure. The USKS showed sound psychometric properties and good convergent validity since it was found to show very strong correlations with the Self-Compassion Scale-Short-Form and the Reassure Self subscale of the Forms of Self-criticizing/Attacking and Self-Reassuring Scale (FSCRS). In addition, the USKS showed good discriminant validity since it was found to show a negative moderate correlation and a negative strong correlation with the HS subscale and with the IS subscale of the FSCRS, respectively. Finally, the USKS showed good test–retest reliability and its use is encouraged in clinical and research settings in which the assessment of a positive attitude toward oneself during an acute presence of threat to the self is of interest.

## 1. Introduction

Western psychology and social sciences have long emphasized the value of a positive attitude toward oneself [1,2,3]. Certain forms of contemplative practices (that are defined by the attentive regulation of breathing), such as compassion-related meditations, may be related to cultivation of positive affect [4,5,6,7,8,9,10]. Specifically, compassion is described by Paul Gilbert and the Buddhist monk Choden as a sensitivity to one’s own and others’ suffering coupled with a commitment to lessen and prevent it [4], while Kristin Neff defines self-compassion as “being open to and moved by one’s own suffering, experiencing feelings of caring and kindness toward oneself, taking an understanding, non-judgmental attitude toward one’s inadequacies and failures, and recognizing that one’s experience is part of the common human experience” [11]. According to recent brain imaging research, compassion is seen as being more emotionally engaging than mindfulness. Positive emotion system areas such the nucleus accumbens, ventral striatum, and medial orbitofrontal cortex have been reported to be activated by compassion practice [12,13,14]. For example, Engen and Singer [15] investigated the effects of compassion meditation with respect to cognitive reappraisal, demonstrating that compassion meditation activated brain systems linked to positive emotions while cognitive reappraisal recruited cognitive control regions and decreased activation of negative affect areas. Studies have also shown that mindfulness practitioners show reduced amygdala activation and structural changes [12,13,14]. Overall, these findings suggest that the therapeutic benefits of mindfulness and compassion may occur via distinct mechanisms: mindfulness may decrease activity in the negative affect system, whereas compassion may enhance function in the positive affect brain networks.

In 2003, the Self-Compassion Scale (SCS) was developed [11]. However, it is not designed to specifically assess self-compassion in situations where being compassionate to oneself may be most difficult and most important. Neff’s [11] scale focuses on the protective factors of self-compassion; however, it does not describe whether people actually apply such protective factors when acutely faced with threats. This issue creates a serious concern because it may be right after a traumatic event, within a peritraumatic time lapse, when it could be most important to be compassionate and kind to oneself [16,17,18]. Conversely, when no threat is active and things are going well, it may be relatively easier to be kind to oneself. Second, despite the six SCS subscales (self-kindness, self-judgment, common humanity, isolation, mindfulness, and over-identification) are informative of important characteristics of compassion and overall well-being, several studies have demonstrated that the subscales are not well correlated. Accordingly, more recent evidence supported a two-factor model of self-compassion with respect to Neff’s original six-factor model [19,20].

According to Buddhism, while compassion meditation focuses on growing goodwill in the midst of suffering [21], loving-kindness (*metta* in Pali) is a state of mind that cultivates unselfish and unconditional kindness to all beings [22] and also involves actively cultivating happiness. Both are constructive practices [21,23] and involve the self as the practice’s object [21,24,25]. Along with loving-kindness (*metta*) and compassion (*karuna*), sympathetic joy (*mutida*; i.e., joy in others’ joy, the opposite of schadenfreude) and equanimity (*upekkha*; being peaceful and well-balanced) represent the four *brahma-viharas*, regarded as the four cultivable sublime states, sometimes referred to as noble and divine abodes or “immeasurables”, as described in the Buddhist text *Visuddhimagga* [26]. Though both loving-kindness and compassion are constructive practices, require the self as the object of practice, and promote the activity of positive emotion brain systems [1], since loving-kindness fosters cultivation of happiness for all beings (including oneself) [22], it may be more directly linked to resourcing and to a positive emotion-focused strategy required to update maladaptive memories [27] and could be more immediately usable in threatening contexts and when dealing with the regulation of peritraumatic emotions. Conversely, since compassion fosters the cultivation of a sensitivity for the suffering of all beings, including oneself, associated to a commitment to alleviate and prevent it [1,4,22], it may be more directly linked to the simultaneous processing and integration of negative and positive affect that may be more demanding to immediately implement during threatening events and when dealing with peritraumatic emotions.

Accordingly, the Unconditional Self-Kindness Scale (USKS) was introduced by Smith et al. [28] as a tool to measure the behavioral response of self-kindness during an acute presence of threat to the self and not just as a general attitude when threat is absent. Since it can be experienced even in the most challenging situations and may promote resilience, this kindness may be defined as unconditional [29]. According to the literature, there are two basic types of kindness: (1) active kindness, such as love and kindness, and (2) passive kindness, such as patience and tolerance [30,31,32]. In addition, the literature has highlighted three kinds of threats in which practicing self-kindness can be difficult but crucial: (1) failure or making mistakes, (2) criticism and rejection, and (3) developing awareness of one’s own imperfections and flaws (e.g., [33,34,35]).

No self-report tools are available in Italian aimed at specifically measuring unconditional self-kindness. Constructs that are related to self-kindness are self-compassion and self-reassurance [4]. Initially, in order to measure self-compassion, the SCS ([11]; Italian version in Veneziani et al. [36]) and the SCS-Short Form (SCS-SF, [37]; Italian version in Poli et al. Submitted) were developed. Self-compassion was defined as being gentle and empathetic toward the self in the face of suffering or failure as opposed to being severely self-critical; seeing one’s own life as belonging to the larger human experience as opposed to seeing them as being unique; and retaining unpleasant thoughts and sensations in attentive awareness instead of over-identifying with them [11]. When faced with challenges, self-reassurance is the capacity to be self-validating, encouraging, sympathetic, to recall one’s positive characteristics, and be reassuring to self when things go wrong [38]. Self-reassurance is linked to increased coping skills, resilience, and persistence [38,39,40,41] and neural markers of negative emotion are down-regulated during attempts to be reassuring to one’s suffering [42,43]. In order to assess self-reassurance and to distinguish self-reassurance from different forms of self-criticizing, the Forms of Self-criticizing/Attacking & Self-Reassuring Scale was developed (FSCRS, [38,44]; Italian version in Poli et al. Submitted). The FSCRS consists of three factors: inadequate self (IS), hated self (HS), and reassure self (RS) [44]. A variety of positive physiological processes and psychological well-being outcomes are linked to self-reassurance and to compassionate attitude towards oneself and others [45]. For instance, there is mounting evidence that, in contrast to self-criticism, supporting, validating, and compassionate approaches to the self are beneficial through several neurophysiological mechanisms [46]. Compassion training may affect processes such as telomere length, which are chromosomal portions that are a biological markers of aging, as well as physiological measures of well-being [47].

In light of the absence of a validated Italian tool with sound psychometric properties aimed at assessing unconditional self-kindness, the aim of our article is to validate the USKS and investigate its psychometric properties [28] in order to contribute a helpful tool that can be used in both clinical and research settings [48,49,50]. The following goals are specifically targeted by the current study: (a) examine the USKS face and content validity; (b) examine the factor structure and psychometric properties of the USKS; (c) investigate the USKS internal consistency and its convergent validity with the SCS-SF ([37]; Italian version in Poli et al. Submitted) and with the RS subscale of the FSCRS ([38]; Italian version in Poli et al. Submitted); (d) investigate its discriminant validity with the IS and HS subscales of the FSCRS ([38]; Italian version in Poli et al. Submitted); and (e) investigate the USKS test–retest reliability.

## 2. Materials and Methods

### 2.1. Participants

The sample consisted of 332 (80.12% female) community participants (M = 43.79 years, SD = 11.42, range 19–76) who responded to email advertisement requesting volunteers for completing psychological questionnaires. The participants’ levels of education were as follows: 80.42% showed the highest level of education (specialization or Ph.D.), followed by 15.36% who had higher-level degrees (bachelor’s or master’s degrees), and 4.22% who had a medium level of education (high school degree). The majority of participants (81.63%) were recruited and working, followed by 3.31% of undergraduate university students, and 15.06% of housewives, unoccupied, and in retirement. In terms of marital status, 33.73% of participants were single, 58.73% of people were cohabiting or married, 6.93% of people were divorced, and 0.6% of people were widows or widowers. 

#### Measures

*Unconditional Self-Kindness Scale* (USKS, [28]). The USKS is a self-report tool aimed to assess unconditional self-kindness developed by Smith et al. [28]. Items invite participants to rate on a 7-point scale (from 0 = Never to 6 = A great deal) their level of agreement with the questions asked in the 6 items (e.g., “How much are you loving and kind to yourself when you are criticized or rejected by another person?”, “How much are you patient and tolerant with yourself when you become aware of your personal flaws and imperfections?”).

Through a combination of forward and back-translation, the Italian version of the USKS was finalized [51]. The English version of the scale was separately translated into Italian by the authors and one psychologist who is multilingual in Italian and English. Following the achievement of translators’ consensus, this Italian-translated version was then translated back into English by an Italian-English researcher who was not aware of the original language. Differences highlighted by this back-translation were addressed with the scale’s authors. Ahead of being utilized in this investigation, the Italian version of the USKS was provided to 10 individuals (not included in the current study) in order to test the items’ readability. All of the questions were determined to be simple to comprehend and to rate. There are different reports about the acceptable values of the Cronbach’s alpha (α, [52]), ranging from 0.70 to 0.95 [53,54,55], as well as recommendations that a very high value (i.e., α > 0.95) may actually be undesirable when developing a test [56]. In our study USKS was found to show an α = 0.932.

*Self-Compassion Scale-Short Form* (SCS-SF, [37]). The SCS-SF is a measure of self-compassion defined as the ability to hold one’s feelings of suffering with a sense of warmth, connection, and concern [37]. It comprises 12 items and 6 subscales: self-kindness, self-judgment, common humanity, isolation, mindfulness, overidentification. Participants are invited to rate on a 5-point scale (from 1 = Almost never to 5 = Almost always) how they typically act towards themselves in difficult times (e.g., “I try to see my failings as part of the human condition”, “When I fail at something that’s important to me, I tend to feel alone in my failure”). In this study we used the Italian version by Poli et al. (Submitted). In our study SCS-SF was found to show an α = 0.885.

*Forms of Self-criticizing/Attacking & Self-Reassuring Scale* (FSCRS, [38]). The FSCRS is a list of 22 items that are aimed at asking the participants to rate on a 5-point scale (from 0 = Not at all like me to 4 = Extremely like me) how much each statement is true for themselves when things go wrong in their lives or do not work out as they had hoped. Nine items are related to the “Inadequate self” subscale (e.g., “I feel beaten down by my own self-critical thoughts”), 5 items are related to the “Hated self” subscale (e.g., “I have become so angry with myself that I want to hurt or injure myself”, “I have a sense of disgust with myself”), and 8 items are related to the “Reassure self” subscale (e.g., “I am able to remind myself of positive things about myself”, “I find it easy to forgive myself”). The Italian version by Poli et al. (Submitted) was used in our study. The IS subscale showed an α = 0.887, the HS subscale showed an α = 0.705 and the RS subscale showed an α = 0.875.

### 2.2. Procedure

Using a safe web-based survey application, the questionnaires were made accessible online (SurveyMonkey). Batteries of questionnaires took between 15 and 25 min to complete. Questionnaires were provided through a balanced method to account for order and sequence effects. The Ethical Principles of Psychologists and Code of Conduct were followed in the treatment of all participants, who voluntarily agreed to participate in the research after being given a thorough explanation of the process [57]. In order to take part in this research, no rewards were provided.

### 2.3. Statistical Analyses

All basic statistical analyses were performed with SPSS^®^ 27 (IBM Corp., Armonk, NY, USA), SigmaPlot^®^ 14 (Systat software, Chicago, IL, USA), AMOS^®^ 27 (Analysis of MOmentum Structures; IBM Corp., Armonk, NY, USA), and Mplus 8.8 [58,59]. As a first stage in the analysis, we investigated the item score distributions to analyze the score frequency distributions of the scores for every item. Specifically, we inspected if each value of the response scale had been adopted at least once, and we also evaluated the amount of the missing data. To verify the normality of the distributions, the Shapiro–Wilk test was used [60].

We examined the data matrix determinant, Bartlett’s test of sphericity, and Kaiser–Meyer–Olkin (KMO) test for sampling adequacy as factorability measures. The full dataset was then used to evaluate the existence of redundant information (too strongly intercorrelated pairs of items) and items with low squared multiple correlations (SMC). These items may identify factors of low relevance in factor analysis, known as “bloated specifics” ([61], p. 288), which are typically generated by very strongly correlated items that often show very similar subject and/or phrasing. Items with an intercorrelation higher than |0.707|, or more than 50% of the shared variance, were deemed redundant. The remaining items’ SMCs were then examined. SMC, or the share of variance that each item participates in with the others, is often employed by EFA software to assess initial communality, or the share of variation that each item’s common factors explain. Items with SMC lower than 0.10 should be eliminated from the item pool since they are unlikely to play a significant role in the measurement model [62].

Hence, a random split of the total sample was applied. Before performing EFA on the first random subsample, we first evaluated the optimal number of components to be extracted using dimensionality measures, such as the scree-test [63], the parallel analysis (PA, [64]), and the minimal average partial (MAP) correlation statistic [65]. The eigenvalues’ curve’s downward trajectory tends to flatten out as the number of factors increases. According to the scree-test, the ideal number of factors corresponds to the eigenvalues’ curve inflection point [63]. In a PA approach, the eigenvalues obtained from a simulated matrix of random data of the same size are contrasted with the eigenvalues observed. In accordance with Buja and Eyuboglu’s [66] guidelines, we carried out PA using 1000 random correlation matrices generated by permuting raw data, and, according to Longman et al. [67], the threshold values used were the 95th percentile random-derived eigenvalues. According to Velicer [65], after partialling out the factors, the number of factors is optimal when the average partial correlation of the variables (i.e., the MAP statistic) is at its lowest value.

Once the optimal number of factors had been established, exploratory structural equation modeling (ESEM, [68]) could be used to evaluate how good the fitness of the models to the data was. Theta parameterization, GEOMIN rotation, and weighted least squares with means and variance adjustment (WLSMV) estimate were used. Overall, ESEM permits the investigation of complex factor structures (similar to EFA) and the estimation of all factor loadings (subject to the identification constraints) while providing access to goodness-of-fit (GOF) indices, parameter estimates, standard errors, and modeling flexibility that are typical features of confirmatory factor analysis (CFA). The final model was chosen based on the GOF indices and the best approximation of a simple structure, applying the same criteria as indicated above for the CFA. We regarded as significant those loadings whose 95% confidence interval was totally above the |0.32| criterion, considering the opportunity provided by ESEM to estimate the standard errors of loadings. We utilized the data of the measurement model to assess its fit through CFA, after identifying it through ESEM. We tested several models in addition to the one-factor model we derived. Another parsimonious model was a bifactor model.

We then tested on the second random subsample through CFA using the WLSMV estimator (theta parameterization) whether the hypothesized one-factor structure was supported by the data at hand. Using the comparative fit index (CFI), the Tucker–Lewis index (TLI), and the root mean square error of approximation (RMSEA) with its 90% confidence interval (CI), the goodness-of-fit was assessed. In order to evaluate the model fit, we used the following guidelines [69]: TLI and CFI values ≥ 0.90 indicated acceptable fit, values ≥ 0.95 indicated excellent fit; RMSEA values ≤ 0.08 indicated acceptable fit, values ≤ 0.06 indicated excellent fit.

By calculating Spearman correlation coefficients between the observed USKS scores and the other measures provided to the sample of participants, construct validity was tested. Following Cohen’s [70] guidelines, correlations between 0.70 and 0.89 were considered very strong, correlations between 0.50 and 0.69 were considered strong, correlations between 0.30 and 0.49 were considered moderate, and correlations between 0.10 and 0.29 were considered weak. 

The first step in evaluating reliability was to perform test–retest analysis of the scales on the second sample of subjects. The Spearman correlation of observed scores at times 1 and 2 (after 3 weeks) was calculated as the retest coefficient. The intraclass correlation coefficient (ICC) was computed to evaluate the stability of scores. We expected retest coefficients greater than 0.70 (i.e., at least 50% of shared variance) in order to detect indications of satisfactory score stability.

Specifically, regarding ICC, we followed the conservative standards that have been proposed by Portney and Watkins [71]: values between 0.5 and 0.75 are considered as “poor to moderate”, values between 0.75 and 0.9 are considered as “good”. We calculated and provided 95% confidence intervals and estimates of effect size wherever it was possible. An additional measure of reliability was assessed through a split-half method, and the two-part Cronbach’s α, the Spearman–Brown coefficient, and the Guttman’s Lambda 4 coefficient were computed [72,73].

Content validity was measured through Cohen’s *K* statistic, assessing inter-rater reliability [74]. The importance of rater reliability lies in the fact that it represents the extent to which the data collected in the study are correct representations of the variables measured. The USKS items were classified by two independent investigators (A.P. and M.M.). The Cohen’s *K* statistic was calculated to determine the inter-rater agreement. The standards proposed by Fleiss, Levin, and Paik [75] and Cicchetti [76] were applied to evaluate whether the value for the *K* statistic is fair (between 0.4 and 0.59), good (between 0.60 and 0.74) or excellent (more than 0.74). The coefficient was excellent (*k* = 0.84). The face validity [77] was evaluated by a convenience sample of 10 students. Items found to be unclear were indicated, and participants were requested to propose a better formulation. Comments were then discussed in the research team until consensus was reached and a final version of the instruments was established.

In order to control for possible gender differences regarding participants’ responses, measurement invariance of the scale’s items across gender was assessed according to recognized recommendations for establishing measurement invariance of models [58,78,79,80]. For the invariance of the measurement parameters, we performed hierarchical tests. As a starting point, we looked at the configural invariance model, also known as pattern invariance, which does not place any limits on model parameters’ equality, including gender in this research. This is a prerequisite for determining invariance via comparison to other invariance models based on fit indices. Second, we looked at the metric invariance model (or weak invariance model). The factor loadings in this model are regarded as being invariant across gender. In order to carry out valid comparisons, this makes sure that the measurements are regarded as having the same scale for both genders. Third, the strong invariance model was considered. Both factor loadings and item intercept must be invariant across gender in this model. This condition ensures that the underlying factors may be compared between genders. In our fourth analysis, we considered the strict invariance model, which requires that the factor loadings, intercepts, and residual variances be invariant across gender. This condition is considered to control for gender invariance in the variances of the regression equations for each item. Evidence of invariance between the more restrictive model (e.g., weak measurement invariance models) and the less restrictive model (e.g., configural invariance model) was based on suggestions from the literature [78,81]. The hypothesis of invariance should not be rejected if the change in CFI (ΔCFI) is ≤ 0.01. The critical values for ΔRMSEA and ΔTLI are 0.015 and 0.01, respectively. For each comparison, the Δχ^2^ was also shown.

## 3. Results

Factorability measures were found to be adequate in order to perform exploratory analyses (determinant = 0.007; Bartlett’s test of sphericity: approximate χ^2^(15) = 1649.533, *p* < 0.0001; KMO test = 0.874). As a first step in the analyses, we examined the item score distributions. The percentage of missing answers never exceeded 1%, the item distribution was not skewed (see Table S1 in the Supplementary Materials), and we planned to analyze these data as ordinal. Since we had to investigate the most adequate measurement model for the Italian USKS without the support of prior knowledge, we decided to perform an EFA on the first random subsample in order to find a factor structure that could meet the requirements of an approximate simple structure [62,82] and a CFA as a subsequent step on the second random subsample. However, we first used the full dataset to identify redundancies and items with low SMC before carrying out these analyses. Items whose intercorrelation exceeded |0.707| (i.e., more than 50% of shared variance) were regarded as redundant. No item exceeded this threshold. Items with SMCs less than 0.10 are not likely to exert a significant contribution to the measurement model and may be eliminated from the item pool [62]. No item was found to show a SMC that was under this threshold.

**Figure 1:**
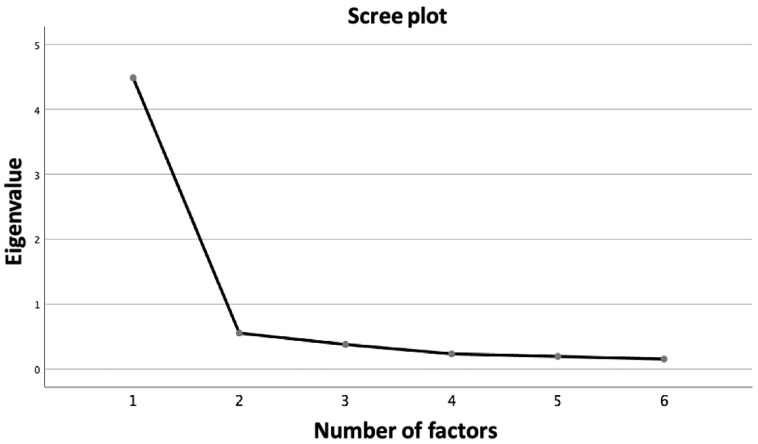
Results of the dimensionality analyses on the first random subsample (*n* = 153).

On the first random subsample, the dimensionality analyses we performed were the scree-test [63] and the PA [64], and we computed the MAP correlation statistic [65]. At the first factor, the scree-plot line seemed to flatten out, indicating the extraction of one or two factors, respectively (Figure 1), and the PA showed that two observed eigenvalues were bigger than the 95th percentile of the associated random eigenvalues. However, The MAP statistic achieved its lowest at the first factor (0.0110, 0.0111, 0.0112, 0.0114, 0.0121, 0.0127). Thus, it became apparent that one or two factors may be the optimal number.

We then used ESEM [68] to test the fit of these models through WLSMV estimation, theta parameterization, and GEOMIN rotation. The results of the ESEM models are reported in Table S2. The bifactor ESEM model showed a poor fit χ^2^(5) = 405.089, *p* < 0.001; CFI = 0.665, TLI = 0.767, RMSEA = 0.285 [0.249; 0.357]. Since we could identify at least three items per factor that had a single loading with a confidence interval fully over 0.32, the one-factor solution had an appropriate fit (χ^2^(5) = 80.776, *p* < 0.001; CFI = 0.931, TLI = 0.933, RMSEA = 0.022 (0.031; 0.037)) and proved to be the most suitable measurement model. The six items loaded on a single factor (0.744, 0.842, 0.848, 0.845, 0.861, 0.869) are shown (Table 1).

**Figure 2:**
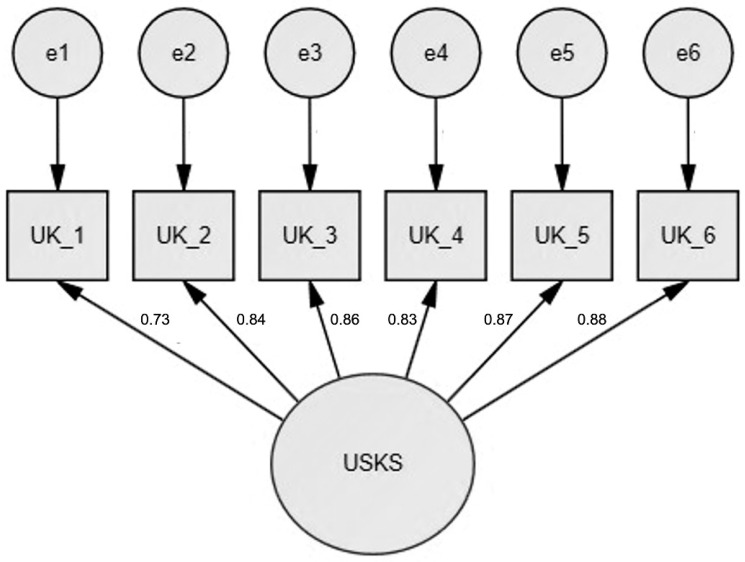
Confirmatory Factor Analysis model for the one-factor solution (*n* = 179). Values above arrows indicate factor loadings. UK = Unconditional Self-Kindness item; e = item error; USKS = Unconditional Self-Kindness Scale factor.

We then tested on the second random subsample through CFA using the WLSMV estimator (theta parameterization) whether the hypothesized one-factor, or bifactor, structures were supported by the data at hand. The goodness-of-fit was evaluated using the CFI, the TLI, and the RMSEA with its 90% CI. We used the aforementioned criteria related to TLI, CFI, and RMSEA for model fit [69] (Table 2). The results confirmed a poor fit of the bifactor model (χ^2^(9) = 750.165, *p* < 0.001; CFI = 0.55, TLI = 0.251, RMSEA = 0.499 (0.469–0.529)) and an adequate fit for the one-factor model (χ^2^(9) = 149.585, *p* < 0.001; CFI = 0.915, TLI = 0.958, RMSEA = 0.033 (0.065; 0.072)) (Figure 2). The six items showed adequate loadings (0.731, 0.849, 0.868, 0.835, 0.879, 0.882) (Table S3).

Table 3 shows the correlations of the scores on the USKS with the other scales in this study. The Italian version of the USKS (Table S4) showed very similar, very strong correlations with the SCS-SF and the RS subscale of the FSCRS, suggesting that higher scores on unconditional self-kindness are associated with a higher tendency to experience self-compassion and self-reassurance, supporting the convergent construct validity of the scale. Conversely, the USKS scores were found to show a negative moderate correlation with the HS subscale of the FSCRS and a negative strong correlation with the IS subscale of the FSCRS, suggesting that higher scores on unconditional self-kindness are associated with a lower tendency to experience negative feelings and thoughts about the self. Taken together, these results seem to support the convergent and discriminant validity of the USKS scale.

We then tested the test–retest reliability of the scale on another sample of participants. The results are reported in Table 4 and show that the scores were fairly consistent in a 3-week period, as test–retest correlation was 0.74. We also carried out the intraclass correlation coefficient (ICC) that confirmed the consistency of the scores in a 3-week period (ICC = 0.845 (0.83; 0.89), *p* < 0.001). In addition, we assessed reliability through a split-half procedure and found that the two split-half Cronbach’s α were acceptable (split-half 1′s α = 0.838; split-half 2′s α = 0.873), as were the Spearman–Brown’s coefficient (C_SB_ = 0.961) and the Guttman’s Lambda 4 coefficient (G_L4_ = 0.96).

We controlled for measurement invariance across gender. The data were well-fit by the configural invariance model (Table S5). The more restrictive measurement invariance (or weak measurement invariance) model that was considered was compared to the configural model. The data were well-fit by the weak invariance model. When the configural invariance model was compared to the weak invariance model, the changes in CFI, TLI, and RMSEA fell within acceptable values (ΔCFI = 0, ΔTLI = 0.001, and ΔRMSEA = 0). These results suggest that the factor score metric was invariant across gender. In other words, the items that were used to assess the factor loadings have the same meaning for both men and women. The strong invariance model, the third increasingly restrictive model, revealed a good data fit as well. This third strong invariance model constrained the factor loadings and item intercept (ΔCFI = −0.003, ΔTLI = −0.002, ΔRMSEA = 0.004). These results suggest that both item intercept and factor loadings are invariant across gender. The fourth, strict invariance model was then analyzed constraining factor loadings, item intercept, and residual variances. Modifications of fit indices fell within the recommended ranges (ΔCFI = −0.004, ΔTLI = −0.004, ΔRMSEA = 0.002). These results show that comparisons of average item scores between males and females are valid.

## 4. Discussion

The aim of the present study was to validate the Italian version of the USKS [28], evaluating the possibility of collapsing response categories, item reduction, its factor structure, reliability, and convergent and discriminant validity. Our results did not support the collapsing of response categories and item reduction. Furthermore, a one-factor structure was confirmed, and the test–retest reliability and the convergent and discriminant validity of the scale were supported. Our results supported a one-factor structure that is in line with the original findings by Smith et al. [28]. The scree-plot was found to show one inflection point and suggested the extraction of one factor. Following guidelines for the acceptance of minimum saturation levels, for newly developed items, saturation per item is suggested to be greater than 0.5, while for established items, saturation for each item is suggested to be 0.6 or higher [83]. Our results fit with this criterion, since all factor loadings are >0.744. Results of the CFA confirmed an adequate fit for the one-factor model following the criteria suggested by Marsh et al. [69]. 

Regarding Spearman correlations and convergent validity, we found very strong positive correlations between the USKS and the SCS-SF and between the USKS and the RS subscale of the FSCRS. In accordance with this, the Dalai Lama [84] highlights that just as compassion is the wish that all sentient beings be free of suffering, loving-kindness is the wish that all may enjoy happiness. Hence, compassion may often involve kindness, but kindness does not need to include suffering and compassion as reported by Gilbert et al. [85]. Thus, as shown by the very strong positive correlation that we found, a strong overlap may exist between unconditional self-kindness and self-compassion, but differences may exist as well. An important difference may be represented by the fact that unconditional self-kindness does not need to include suffering and self-compassion, and it may be a useful attitude to promote during the most challenging situations. Analogously, considering the very strong positive correlations between the USKS and the RS subscale of the FSCRS, a strong overlap may exist between unconditional self-kindness and self-reassurance as well. Self-reassurance, has been defined as the ability to be soothing, encouraging, and supportive to oneself in the face of setbacks [38,86], while loving-kindness has been defined as a state of mind that cultivates unselfish and unconditional kindness to all beings and entails intentionally cultivating happiness [22]. However, some differences may exist between unconditional self-kindness and self-reassurance. In accordance with this, it has been shown that self-reassurance did not activate regions such as the insula, anterior cingulate cortex, and amygdala which are typically active during self-criticism [42], while it was found that loving-kindness practice increased the neural responses of the dopamine system (ventral tegmental area and orbitofrontal cortex) and increased self-reported positive affect as well [87]. In addition, it has been proposed that happiness, that is intentionally cultivated during loving-kindness, may represent an immediate emotional experience—a feeling that relies on the neurophysiological activation of the dopaminergic brain’s reward system [88]. Overall, these findings suggest that self-reassurance may promote a reduction of the negative affect system’s activity while unconditional self-kindness may promote an increase of the positive affect system’s activity. Interestingly, this pattern of neural activity related to unconditional self-kindness and self-reassurance parallels the pattern of neural activity observed for mindfulness and self-compassion: mindfulness was suggested to reduce the negative affect system’s activity while compassion was suggested to increase activity of positive emotion brain systems [1]. In accordance with these findings, regarding discriminant validity, we found a strong negative correlation between USKS and the IS subscale of the FSCRS and a moderate negative correlation between USKS and the HS subscale of the FSCRS. In fact, self-criticism, but not self-reassurance, activated regions of the negative affect system such as the insula, anterior cingulate cortex, and amygdala [42,43,89].

Concerning the test–retest reliability of the USKS, we examined it on another sample of participants that answered again after 3 weeks. The USKS scores obtained were consistent after a 3-week period: in fact, the Spearman test–retest correlation was above 0.70; in addition, according to the conservative guidelines suggested by Portney and Watkins [71], ICC was found to be above 0.80. Overall, these results indicated that the USKS showed a good test–retest reliability. Furthermore, using a split-half method, we found that the two split-half Cronbach’s α, the Spearman–Brown’s coefficient, and the Guttman’s Lambda 4 coefficient were all above 0.80, confirming that the USKS retained a good test–retest reliability. Eventually, measurement invariance analyses across gender confirmed that comparisons of average item scores between males and females are valid, ruling out the possibility of a gender bias.

## 5. Limitations and Conclusions

The findings of this study should be interpreted in light of some limitations: (a) first, the psychometric properties of the scale were examined in a large non-clinical sample derived from the general Italian population; additional research is needed to confirm the scale’s one-factor structure and adequate reliability and validity in clinical samples; (b) the participants’ demographics were not representative of the general population, and it may limit the generalizability of the findings; (c) two of the measures utilized in the study do not yet have an Italian published validation, since the articles are in preparation or in the process of being published; and (d) we did not evaluate criterion and nomological validity that should be assessed in future studies.

Although more research is required to replicate these results and validate the measure in languages other than Italian before it can be confidently used in clinical and research settings where this construct is of interest, this study provides preliminary evidence that the USKS is a reliable unidimensional scale to assess unconditional self-kindness.

## Figures and Tables

**Table 1:** Loading matrix and factor correlations of the one-factor Exploratory Structural Equation Modeling solution on the first random subsample (*n* = 153).

Item	F1
USKS01	0.744 [0.22; 0.44]
USKS02	0.842 [0.23; 0.44]
USKS03	0.848 [0.32; 0.51]
USKS04	0.845 [0.37; 0.58]
USKS05	0.861 [0.51; 0.68]
USKS06	0.869 [0.27; 0.51]

Note: Bracketed values and the 95% confidence interval of the loading estimate.

**Table 2:** Goodness-of-fit indices for the Confirmatory Factor Analyses on the second random subsample (*n* = 179).

Model	χ^2^	df	CFI	TLI	RMSEA [90% CI]
One-factor	149.585	9	0.915	0.958	0.033 [0.065; 0.072]
Bifactor	750.165	9	0.550	0.251	0.499 [0.469; 0.529]

Note: all chi-square tests were significant at *p* < 0.001; df = degrees of freedom; CFI = Comparative Fit Index; TLI = Tucker–Lewis Index; RMSEA = Root Mean Square Error of Approximation; CI = confidence interval.

**Table 3:** Spearman correlations among USKS and the other study measures (*n* = 332).

Scales	1	2	3	4	5
USKS	*0.932*				
SCS-SF	0.734 ***	*0.885*			
IS	−0.580 ***	−0.809 ***	*0.887*		
HS	−0.351 ***	−0.574 ***	0.657 ***	*0.705*	
RS	0.704 ***	0.746 ***	−0.685 ***	−0.567 ***	*0.875*
M	18.15	3.39	14.56	2.56	21.08
SD	7.58	0.8	7.74	2.67	5.55
Median	18	3.5	14	2	22
IQR	11	1.33	12	3	7

Note: ***: correlations are significant at *p* < 0.001. Italicized values on the main diagonal are Cronbach’s alphas. USKS: Unconditional Self-Kindness Scale; SCS-SF: Self-Compassion Scale–Short Form; IS: Inadequate Self subscale of the Forms of Self-Criticizing/Attacking and Self-Reassuring Scale (FSCRS); HS: Hated Self subscale of the FSCRS; RS: Reassure Self subscale of the FSCRS; M: mean; DS: standard deviation; IQR: Interquartile Range.

**Table 4:** Reliability measures for the USKS scale.

Reliability Measure	Test		Retest			
Test–Retest	M (Med)	SD (IQR)	M (Med)	SD (IQR)	ρ_tt_	ICC
	17.29 (17)	8.14 (14)	18.49 (18)	6.62 (8)	0.74 ***	0.845 ***
Split-half	Split-half-1–Cronbach’s α		Split-half-2–Cronbach’s α		C_SB_	G_L4_
	0.838		0.873		0.961	0.96

Note: USKS: Unconditional Self-Kindness Scale; M: mean; SD: standard deviation; Med: Median; IQR: Interquartile range; ρ_tt_: Spearman test–retest correlation; ICC: Intraclass correlation coefficient; α = standardized Cronbach’s α; C_SB_ = Spearman–Brown’s coefficient; G_L4_ = Guttman’s Lambda 4 coefficient; ***: *p* < 0.001.

## Data Availability

The datasets generated during and/or analyzed during the current study are available from the corresponding author on reasonable request.

## Supplementary Materials

The following supporting information can be downloaded at: https://www.mdpi.com/article/10.3390/ijerph20105839/s1, Table S1: Shapiro-Wilk statistics for the item scores distribution of the 6-item; Table S2: Goodness-of-fit indices for the Exploratory Structural Equation Modeling (ESEM) on the first random subsample; Table S3: Loading matrix and factor correlations of the one-factor Confirmatory Factor Analysis model on the second random subsample; Table S4: Italian version of the Unconditional Self-Kindness Scale (USKS); Table S5: Measurement invariance across gender on the total sample.
