# Supplementary material for: Validation and Psychometric Properties of the Italian Version of the Unconditional Self-Kindness Scale (USKS)
Source: Int J Environ Res Public Health. 2023 May 16;20(10):5839. doi: 10.3390/ijerph20105839 (PMC10218580; doi:10.3390/ijerph20105839)
Supplement: Supplementary file 1 [file ijerph-20-05839-s001.zip › Supplementary_Tables.pdf]

Supplementary Materials

Table S1.

Shapiro-Wilk statistics for the item scores distribution of the 6-item USKS (n = 332).

Item	7-point scoring		
	W	df	*p*
usks01	0.989	332	0.075
usks02	0.991	332	0.079
usks03	0.986	332	0.071
usks04	0.987	332	0.073
usks05	0.992	332	0.081
usks06	0.988	332	0.074

Note: W = Shapiro-Wilk statistic; df = degrees of freedom; all Shapiro-Wilk tests showed normal distributions with $p > .05$ and W → 1.

Table S2.

Goodness-of-fit indices for the Exploratory Structural Equation Modeling (ESEM) on the first random subsample (n = 153).

Model	χ^2	df	CFI	TLI	RMSEA [90% CI]
One-factor	80.776	5	.931	.933	.022 [.031; .037]
Bifactor	405.089	5	.665	.767	.285 [.249; .357]

Note: all chi-square tests were significant at $p < .001$; df = degrees of freedom; CFI = Comparative Fit Index; TLI = Tucker Lewis Index; RMSEA = Root Mean Square Error of Approximation; CI = confidence interval.

Table S3.

Loading matrix and factor correlations of the one-factor Confirmatory Factor Analysis model on the second random subsample (n = 179).

Item	F1
USKS01	0.731 [0.23; 0.41]
USKS02	0.849 [0.21; 0.39]
USKS03	0.868 [0.33; 0.49]
USKS04	0.835 [0.36; 0.59]
USKS05	0.879 [0.53; 0.67]
USKS06	0.882 [0.26; 0.48]

Note: Bracketed values and the 95% confidence interval of the loading estimate.

Table S4.

Italian version of the Unconditional Self-Kindness Scale (USKS).

Unconditional Self-Kindness Scale (USKS)

Per favore, risponda a queste domande nel modo più onesto possibile identificando un numero sulla scala seguente:

Per niente						Moltissimo
0	1	2	3	4	5	6

		Punteggio
1	Quanto sei paziente e tollerante con te stesso quando sei criticato o rifiutato da un'altra persona?	
2	Quanto sei amorevole e gentile con te stesso quando diventi consapevole dei tuoi difetti e imperfezioni personali?	
3	Quanto sei paziente e tollerante con te stesso quando fallisci o commetti un errore?	
4	Quanto sei amorevole e gentile con te stesso quando vieni criticato o rifiutato da un'altra persona?	
5	Quanto sei paziente e tollerante con te stesso quando diventi consapevole dei tuoi difetti personali e imperfezioni?	
6	Quanto sei amorevole e gentile con te stesso quando fallisci o commetti un errore?	

Scoring:

Total USKS scoring
Sum of item scoring: 1+2+3+4+5+6

Per niente = 0; Moltissimo = 6

Table S5.

Measurement invariance across gender on the total sample (n = 332).

Number	Model	χ^2	df	CFI	TLI	RMSEA	SRMR	Model comparison	$\Delta\chi^2$	Δdf	ΔCFI	ΔTLI	ΔRMSEA	ΔSRMR
1	Configural	14.773	18	0.998	0.997	0.035	0.043	-	-	-	-	-	-	-
2	Metric (weak)	15.769	23	0.998	0.998	0.035	0.045	2 vs. 1	0.996	5	0	0.001	0	0.002
3	Scalar (strong)	15.769	29	0.995	0.996	0.039	0.045	3 vs. 2	0	6	-0.003	-0.002	0.004	0
4	Strict	16.821	35	0.991	0.992	0.041	0.048	4 vs. 3	1.052	6	-0.004	-0.004	0.002	0.003

Note: χ^2 = chi-square value; df = degrees of freedom; CFI = Comparative Fit Index; TLI = Tucker Lewis Index; RMSEA = Root Mean Square Error of Approximation; SRMR = standardized root mean residual; Δ = delta indicates the difference between values related to model comparisons.
